# Using land use/land cover trajectories to uncover ecosystem service patterns across the Alps

**DOI:** 10.1007/s10113-017-1132-6

**Published:** 2017-03-11

**Authors:** Lukas Egarter Vigl, Erich Tasser, Uta Schirpke, Ulrike Tappeiner

**Affiliations:** 1 Institute for Alpine Environment, Eurac Research, Viale Druso 1, 39100 Bolzano, BZ Italy; 2 Institute of Ecology, University of Innsbruck, Sternwartestr. 15, 6020 Innsbruck, Austria

**Keywords:** Ecosystem services bundles, Spatiotemporal dynamics, ES upscaling, Cluster analysis, Mountain areas

## Abstract

Managing multiple ecosystem services (ES) in agricultural landscapes is a challenging task, especially in regions with complex topographical and agro-ecological conditions. These challenges require ES assessment approaches that go beyond the case study level and provide multi-temporal information at a transnational level. We used a spatiotemporal approach to examine the impact of specific land use/land cover (LULC) trajectories on eight ES for the past 150 years. We show how a spatially explicit ES upscaling procedure, from case study to an Alpine-wide level, based on topographical, agro-ecological and socioeconomic parameters, can improve our understanding of ES dynamics and bundles. Our results indicated that the provision of multiple ES was not stable during the 150 years surveyed, mainly depending on the prevailing land management type and the biophysical conditions. ES bundle mapping enabled us to identify landscapes with consistent socioecological characteristics that are most likely to either enhance or diminish the provision of specific types of services. By introducing a spatiotemporal perspective into ES assessment, we provide clear evidence of the dynamic nature of ES provision and contribute to identifying processes and drivers behind these interactions. Our results emphasize that mountain ES supply is particularly sensitive to long-term LULC change, to biophysical characteristics and to regional socioeconomic conditions. They indicate the benefit of integrating of ES bundles into environmental policies at national and transnational level.

**Electronic supplementary material:**

The online version of this article (doi:10.1007/s10113-017-1132-6) contains supplementary material, which is available to authorized users.

## Introduction

Present on all continents and having some of the richest biodiversity, mountain landscapes contribute to human well-being in many different ways (EEA 2010; Grêt-Regamey et al. 2012). They deliver food and shelter (crops, fodder, water, fuels, materials) (Cooper et al. 2009; Briner et al. 2013), protection and health (prevention of soil erosion, climate regulation, medical plants) (Ruiz-Mirazo et al. 2011; Bernstein 2014) or pleasure (recreational experiences in landscapes, cultural heritage, enjoyment of appealing landscapes) (Sayadi et al. 2009; Lindemann-Matthies et al. 2010; Schirpke et al. 2016). However, they are facing a number of serious and growing challenges because of a variety of human activities: increased urbanization (Wang and Mountrakis 2011); agricultural ex- or intensification (Tasser et al. 2007; Terrasson et al. 2015); increased economic pressure from globalized land management directions (Jepsen et al. 2015); and rapid changes in tourism activities and developments (Geneletti 2008). These practices affect land use and land cover (LULC) and consequently ecosystem services (ES) in various ways. For example, in the European Alps over the past decades, land use in many valley bottoms was intensified, but large areas of mountain grasslands were abandoned and subsequently reforested (Tasser et al. 2007; Rutherford et al. 2008), influencing agricultural services and particularly regulating and cultural services (Briner et al. 2013; Schirpke et al. 2013).

 The ES concept, defined as all the benefits people derive from nature (Costanza et al. 1997), provides a flexible framework for addressing the multiple interdependencies between ecological and socioeconomic systems (de Groot et al. 2002; MA (Millennium Ecosystem Assessment) 2005). Today, the ES framework is widely recognized as a primary instrument to support policy and decision makers in the context of ecosystem conservation (Daily et al. 2009; Honey-Rosés and Pendleton 2013). This development is reflected in different initiatives worldwide, such as the Mapping Ecosystems and their Services (Maes et al. 2013) framework within Action 5 of the European Union (EU) Biodiversity Strategy or The Economics of Ecosystems & Biodiversity (TEEB 2010) process at a global level. These initiatives all have the common objective of providing instruments for the effective preservation and enhancement of biodiversity and ES provision across different biophysical, socioeconomic and political contexts.

To address these targets and to ensure the continued delivery of such crucial ES, sustainable ecosystem management in mountain landscapes requires policy plans that go beyond national borders and include a transnational perspective that is preferably not limited to a single point in time (Egarter Vigl et al. 2016). However, such strategies are usually time-consuming, often requiring labor-intensive fieldwork (i.e., to acquire land use data or to conduct extensive statistical surveys), especially if the studies cover large geographic areas in complex terrain. This has led researchers to include the results from other investigations in their research and to develop methods capable of transferring site-specific findings to other landscape units (Lavorel et al. 2011). These procedures usually involve simple ES transfer functions (Brander et al. 2012) based on LULC, socioeconomic and/or topographic conditions in the study area. An increasing need for ES assessments at large geographic scales is documented in a series of studies around the world, such as Haines-Young et al. (2012) and Maes et al. (2011) for Europe, Lawler et al. (2014) for the USA and Yang et al. (2015) for China. The integration of historical perspectives into ES assessment has essential advantages for stakeholder groups, policy and decision makers, and academics (Rhemtulla et al. 2007): First, it sheds light on human impacts on LULC and related ecosystem functions. Second, it allows the rate of change and the underlying causes of ES dynamics over a temporal component to be determined. Third, the rate of changes can be used as a proxy for estimations of possible future ES behavior.

Recent studies have assessed ES at different spatial scales, making use of geographic information system (GIS) technology to facilitate the understanding of ES provision and patterns (Grêt-Regamey et al. 2014; Rabe et al. 2016). Analyzing the spatiotemporal interactions among multiple ES allows the identification of multiple ES provision occurring either in synergy or as trade-offs within specific landscapes (Rodriguez et al. 2006). It is possible to group ES values into so-called bundles through statistical analysis (Bennett et al. 2009; Raudsepp-Hearne et al. 2010) and to assess their temporal behavior over broad time periods (Renard et al. 2015). In the face of the globally observed increasing pressure from anthropogenic activities on natural systems, extensively documented in several studies on long-term LULC trajectories (Gimmi et al. 2010; Lambin and Meyfroidt 2011; Meyfroidt et al. 2013; Levers et al. 2015), understanding of how such ES bundles form and behave becomes crucial (Raudsepp-Hearne et al. 2010). Knowing whether the presence of a specific ES bundle excludes the presence of another bundle or identifying ecosystems where multiple services are likely to coexist, will be an essential advantage for landscape management and for promoting the importance of landscape functions to policy makers and society (Crouzat et al. 2015). Some of the most significant studies in recent years in this context include ES pattern analysis in Denmark by Turner et al. (2014), spatiotemporal analysis of ES bundles at the municipality level by Renard et al. (2015) in the greater Montreal region (Canada) and the analysis of Queiroz et al. (2015) on the multi-functionality of Swedish landscapes. All these studies aim to identify ES patterns based on the underlying socioecological subsystems and the specific LULC composition. They clearly show that it is crucial to develop approaches that allow trans-border assessments of ES and their trade-offs, enhance the capabilities for ES clustering into bundles and eventually provide the basis for a sustainable balance between ES protection and uses.

Studies focusing specifically on mountain landscapes are under-represented in this context. Most published work in alpine regions concentrates either on a single case study (Schirpke et al. 2013; Bürgi et al. 2015a) or on a single point in time (Lamarque et al. 2011; Grêt-Regamey et al. 2014; Crouzat et al. 2015). To our knowledge, only the work carried out by Egarter Vigl et al. (2016) (subsequently referred as the Alpine Study) aimed to combine transnational and temporal components into a single study on mountain regions, while keeping the focus at a case study level. The main objective of this paper was to draw on the findings of the Alpine Study and to integrate them with: (1) an ES-mapping approach that used distinct types of LULC trajectories (Zimmermann et al. 2010) to transfer ES trend values to an alpine-wide level and (2) to perform a geostatistical cluster analysis for the spatiotemporal identification and interpretation of ES bundles through time. We discuss how such a spatiotemporal ES assessment approach for eight ES over broad time periods (past 150 years) and across national borders can help us to improve our understanding of ES dynamics, their protection, sustainable use, management and risk prevention in complex mountain landscapes.

## Methods

**Fig. 1:**
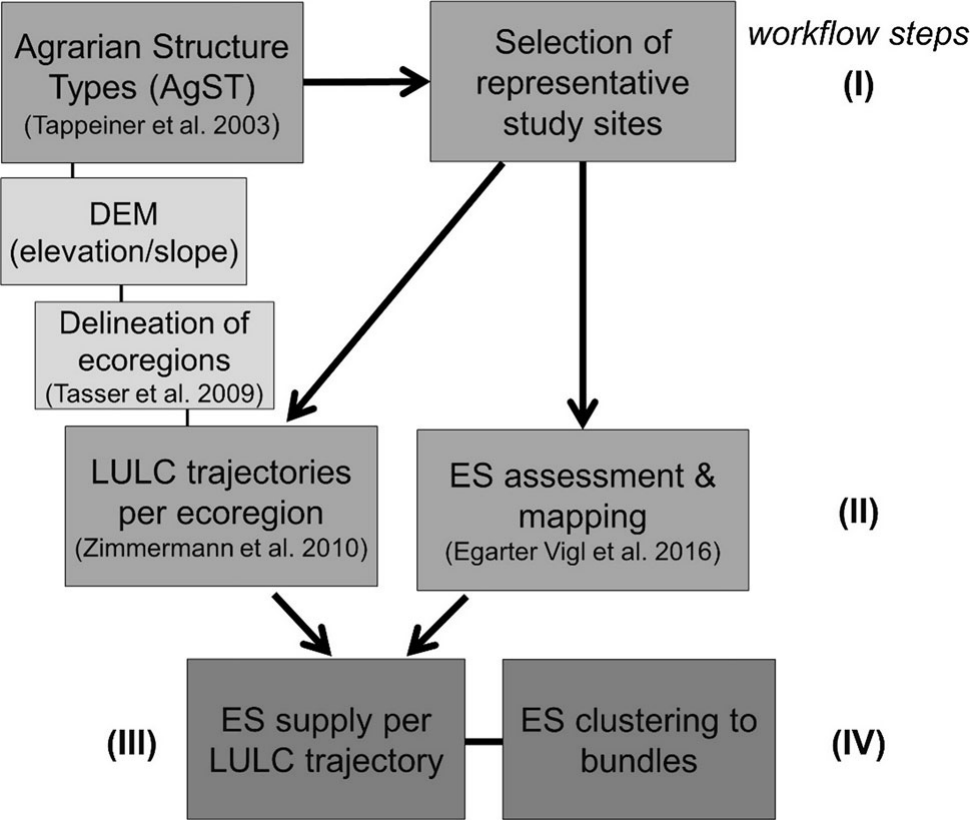
Flowchart depicting the procedures used to derive ES trend maps and bundles. *Numbers* indicate the workflow sequence, while the gradient in the *gray scale* of the boxes (from *light* to *dark gray*) indicate the *core parts* of the present study

The methodological approach applied in this paper comprised four steps: (I) the definition of specific case study sites across the European Alps, based on their representativeness for the main Agrarian Structure Types (AgST); (II) the spatiotemporal assessment of multiple ES at the ecoregion level; (III) the allocation of ES capacities to LULC trajectories to derive continuous ES trend maps; and (IV) the geostatistical clustering analysis for the identification of ES bundles (Fig. 1).

### Case study selection and definition of ecoregions

**Fig. 2:**
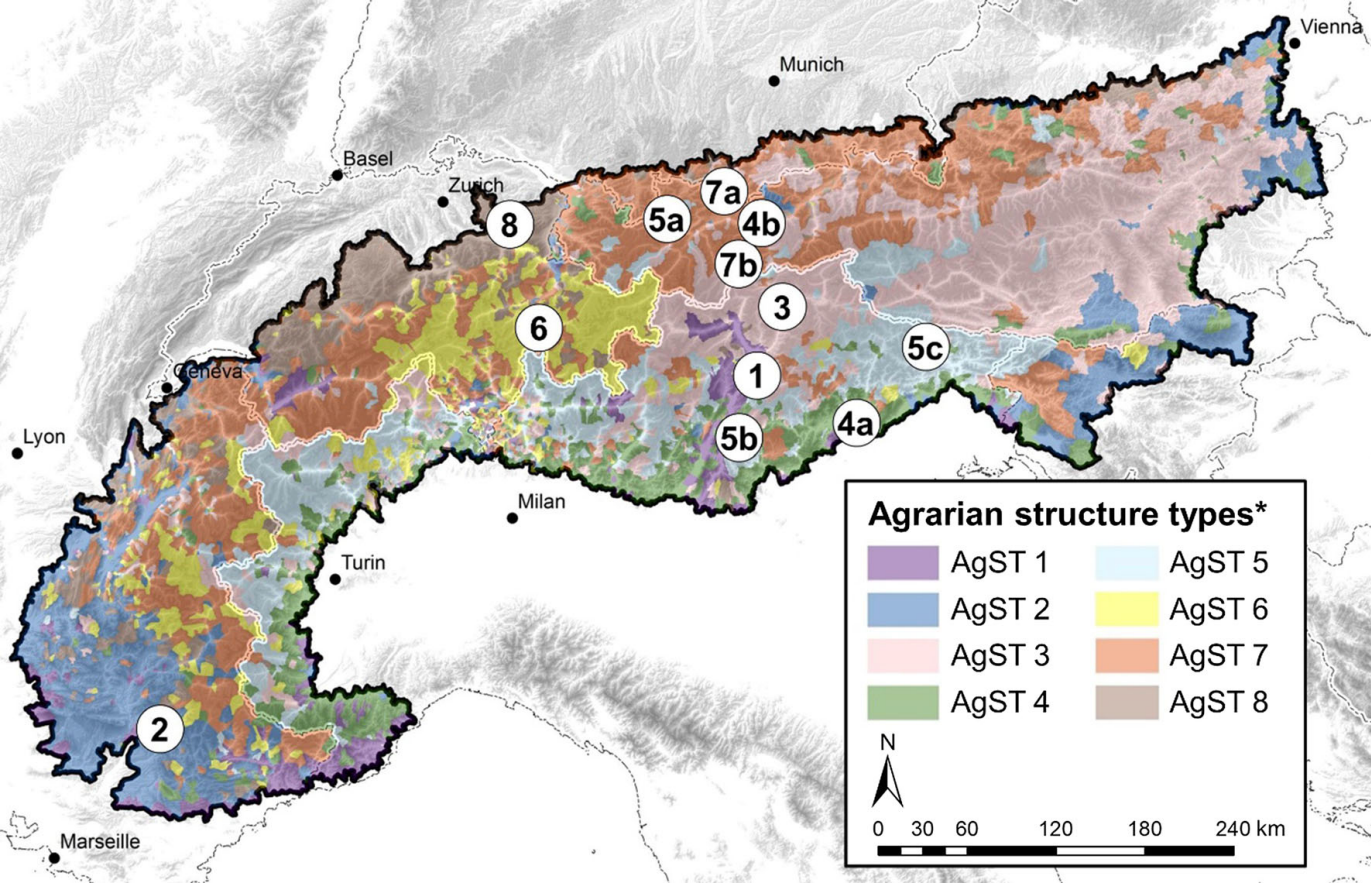
Location of the case study sites. (*1*) Unterland, (*2*) Alpes-de-Haute-Provence, (*3*) South Tyrolean Mountains, (*4a*) Piave, (*4b*) Innsbruck, (*5a*) Lechtal, (*5b*) Trentino Mountains, (*5c*) Carnia, (*6*) Graubünden, (*7a*) Garmisch-Partenkirchen, (*7b*) Stubai-Tyrolean mountain region, (*8*) Toggenburg. Colored areas depict AgST according to Tappeiner et al. 2003. *Agrarian Structure Types according to Tappeiner et al. (2003)

To perform our analysis, we used datasets mainly originating from the Alpine Study. However, we integrated them with a substantial number of new municipalities, to increase their representativeness for the eight main Agrarian Structure Types (AgSTs) present in the Alps (Tappeiner et al. 2003, 2008). AgSTs describe regions with a common range of environmental, agro-economic and political conditions, formed by a statistical clustering process on 43 indicators in more than 6000 municipalities. Subsequently, we selected at least one group of municipalities around each cluster-centroid for deeper analyses. In total, 57 municipalities distributed across 12 study sites were selected (Fig. 2), covering approximately 3400 km^2^ or ~2% of land mass in the Alps.

To account for the ES changes that can occur within single case studies with specific topo-climatic conditions, each site was further subdivided into ecoregions according to Tasser et al. (2009). Ecoregions denote landscape units with similar ecosystem properties that originate through the interaction of site characteristics, agricultural/silvicultural use, and settlement and infrastructure development. In the present study, the ES trend analyses were executed at the scale of these ecoregional subdivisions. The use of these ecoregions helps highlighting the broader historical context of the landscape by allowing different study sites to be compared by topographic and land use properties. Ecoregions were delineated using datasets on elevation (altitudinal zone), topographic position (slope) and predominant land use pattern (forest, agriculture or natural) for the year 1850. This early time period was selected for its validity in terms of land use pattern throughout the Alps: forests were considered to be at their overall minimum extension and agricultural land at its maximum (Mather et al. 1999). Similar approaches have also been used successfully in other studies (Loveland et al. 2002). Overall, we identified 10 ecoregions (Table 1) and analyzed each to record and evaluate the changes in ES.

**Table 1:** Subdivision of Alpine landscapes into ecoregions and their occurrence in the analyzed case study sites (after Tasser et al. 2009)

	Ecoregion	Altitudinal zone	Total area (km^2^)	AgST-Study sites							
				1	2	3	4	5	6	7	8
(a)	Vegetation-less belt	Nival	143.2			×				×	
(b)	Near-natural grassland	Alpine	660.0			×	×	×	×	×	
(c)	Agriculturally used alpine pastures	Subalpine/alpine	493.0			×	×	×	×	×	
(d)	Forest belt	Subalpine	659.2			×	×	×	×	×	
(e)	Forest belt	Montane	597.2	×	×	×	×	×	×	×	×
(f)	Agriculturally used valley slopes	Montane	351.3	×	×	×	×	×	×	×	×
(g)	Agriculturally used valley bottom	Montane	228.0		×	×	×	×		×	×
(h)	Forest belt	Colline	84.0	×			×	×			
(i)	Agriculturally used valley slopes	Colline	59.8	×			×				
(j)	Agriculturally used valley bottom	Colline	98.9	×	×		×	×			

### Study site descriptions

We first provide a general characterization for each of the eight AgST, followed by a basic description of the municipalities analyzed in our case studies.

#### AgST 1: High labor, intensive crop region

*AgST 1* sites are characterized by a high percentage of intensive crop cultivation (i.e., fruit, viniculture, vegetables and flowers), a small average farm size and a mild climate. The low percentage of farm abandonments and minor changes in LULC over the past decades suggest a relatively stable agrarian situation. For this region, we selected four municipalities (Leifers, Montan, Kaltern and Kurtatsch) as case studies along the Adige valley in northern Italy *(Unterland)*, covering a total of 120 km^2^ of land.

#### AgST 2: Labor-extensive arable land region

*AgST 2* sites are characterized by a mix of mainly forested slopes and mountain peaks and a climate that has a Mediterranean influence. The above-average farm sizes and a high percentage of farm abandonment, together with a decline in part-time farming and a high percentage of unused agricultural land, indicate that in this region a process of structural adjustment toward specialized, labor-extensive full-time farming is underway. For this AgST, we selected *Alpes*-*de*-*Haute*-*Provence* study site located in the Prealps of southeastern France. It comprised the municipalities of Barras, Hautes Buyes, Mirabeau, and Thoard and covered approximately 107 km^2^ of land.

#### AgST 3: Grassland region

*AgST 3* (Grassland region) sites are characterized by a high employment rate in the agricultural sector, mainly on grazing and livestock farms. Tourism activities, however, are increasingly important and are often economically integrated with specialized livestock farming. We analyzed the *South Tyrolean Mountains (IT)* municipalities of Antholz, Ahrntal, Gais, Glurns, Graun, Gsies, Mals, Percha, Prettau, Ritten, Sand in Taufers, St. Leonhard in Passeier, St. Martin in Passeier, and Schluderns, covering approximately 1311 km^2^ of land.

#### AgST 4: Small-scale grassland farms

*AgST 4* sites are characterized by low mountain ranges and broad valley floors, where the remaining agricultural areas are used intensively. At higher elevations, agriculture has almost ceased. Settlements, mixed deciduous woods and coniferous forests cover large areas. For this AgST, we analyzed the *Piave* site (Fig. 2, 4a), located at the southern border of the southeastern Alps in Italy and the *Innsbruck* site (Fig. 2, 4b) located at the northern border of the Central Eastern Alps in Austria. In total, this study site comprised six municipalities (Limana, Mel, San Gregorio, Santa Giustina, Trichiana and Innsbruck) covering approximately 250 km^2^ of land.

#### AgST 5: High farmland abandonment

*AgST 5* sites are characterized by difficult conditions for agricultural production: a very wet, cool winter climate and a hot, dry summer climate, steep slopes, and usually a very small farm structure. As a result, over the past decade, most agricultural areas have been abandoned and are now covered by forests. In total, we investigated four municipalities in the region of Lechtal (Gramais, Hinterhornbach, Pfafflar, Stanzach) (Fig. 2, 5a), three in the Trentino Mountains region (Lomaso, Terragnolo, Transacqua) (Fig. 2, 5b), and four in the Carnia region (Cercivento, Comeglians, Ravascletto and Sauris) (Fig. 2, 5c). Together, these covered an area of 421 km^2^.

#### AgST 6: Structured, full-time agricultural region

*AgST 6* sites include the characteristics of Swiss agriculture in the Inner-Alpine area: a relatively high employment rate in the agricultural sector and a tendency to agricultural intensification in favorable locations. However, winter tourism is an increasingly important economic factor. The natural condition of the region differs because of its topography. It covers surfaces on terraces (1000–1400 m a.s.l.) as well as Alpine valleys situated between 1500 and 3000 m. The climate is influenced by the ‘dry Inner-Alpine-zone’ with maximum precipitation during summer. For this AgST, we analyzed parts of the *Graubünden* region, comprising the municipalities of Casti-Wergenstein, Mathon, Tschappina and Urmein (70 km^2^ in total) located in the eastern part of the Swiss Alps (Fig. 2, 6).

#### AgST 7: Alpine ‘standard region’

*AgST 7* sites are characterized by landscapes that are dominated by forests and grasslands, which differ in management intensity and include meadows of high land use intensity in the valley bottom, alpine meadows of low land use intensity and pastures at higher altitudes, along with abandoned pastures and meadows. In total, we analyzed two municipalities in Germany (Garmisch-Partenkirchen and Farchant) and six in Tirol (Fulpmes, Längenfeld, Mieders, Mutters, Neustift, Schönberg Telfes im Stubai), covering approximately 567 km^2^ of land (Fig. 2, 7a, b, respectively).

#### AgST 8: Large-scale cattle breeding

*AgST 8* sites represent the characteristics of Swiss agriculture at the northeastern borders of the Alps in transition to the agriculturally favorable areas of the Mittelland region (CH). The elevation gradient typically ranges between 600 and 1000 m a.s.l. and the climatic conditions are humid with a distinct cold season. The prevailing agriculture system is characterized by full-time large-scale cattle breeding farms in favorable locations and is typically labor-extensive. For this AgST, we selected the case study *Toggenburg (CH)* that comprises the municipalities of Brunnadern, Oberhelfenschwil, Krinau and Wattwill and covers approximately 71 km^2^ of land.

### Quantification of ES and spatiotemporal scales

To quantify ES, we followed the approach of the Alpine Study in which five ES (crop production, green biomass, climate regulation, soil erosion protection, aesthetic value) were assessed in biophysical terms, mainly using LULC data from different time periods and sets of socioeconomic census data. Building on the same data pool, we also developed one regulating (pollination potential) and two cultural ES (recreation and mushroom picking potential), resulting in a set of eight ES (Table 2). Pollination was calculated based on an approach by Maes et al. (2011), which assigns each natural ecosystem a specific capacity to support pollination services for nearby cultivation. Recreation potential was calculated as the sum of recreational areas (ha/capita^−1^) within walking distance from human settlements (Larondelle and Hasse 2013). Mushroom picking was estimated using an approach discussed by Schirpke et al. (2014) that identifies all forested areas that meet specific topographic conditions (elevation and slope). A more detailed description of ES assessment methodology and historical data sources is provided in Online Resource 1.

**Table 2:** Ecosystem services assessed for the years 1850, 1955, 1985 and 2005 across the European Alps

Ecosystem service	Indicator	Unit	Calculation method	References
Provisioning ES				
Cultivated crops	Working hours	h ha^−1^	Sum of working hours needed to buy basic agric. commodities of 1 ha of land	Egarter Vigl et al. (2016)
Green biomass	Amount of forage	Mg DM ha^−1^	Quantified for each grassland type as a function of LU-intensity, length of vegetation period, climate, and topography	Egger et al. (2005); Tasser et al. (2012)
Regulating ES				
Climate regulation	Carbon stocks by terrestrial ecosystems	Mg C ha^−1^	Carbon stocks of above and below ground phytomass retrieved from own measurements and literature	Tappeiner et al. (2008); Patek (2013)
Soil erosion control	Erosion rates	Index	Modified USLE	Wischmeier and Smith (1978)
Pollination	Pollination contribution by ecosystems	Index	Capacity of natural ecosystem to provide pollination service as function of distance and ecosystem type	Maes et al. (2011); Klein et al. (2007); Ricketts et al. (2008)
Cultural ES				
Aesthetic value	Landscape beauty	Index	Combination of photo survey, GIS viewshed analysis, and landscape metrics index	Schirpke et al. (2013)
Recreation	Recreational surface per capita	ha capita^−1^	Recreational areas (forests, natural grassland, water) within a distance of 5 km to settlements divided by the number of residents	Larondelle and Hasse (2013); Barbosa et al. (2007)
Mushroom picking	Potential area for mushroom picking	ha^−1^	Forested land cover close to tracks at low elevation and slope angles (<2000 m and <80%, respectively)	Schirpke et al. (2014)

Recreation was not calculated for the year 1850 because it was not considered relevant for this early time period

Each ES was quantified for the years 1850, 1955, 1985 and 2005, roughly covering the most important land management dynamics of the past 150 years in the Alps (Jepsen et al. 2015). The recreational service was excluded from the earliest time period as it was not considered to be a relevant ES in 1850. Between 2005 and the date of publication of this work, LULC has changed relatively little in the study areas due to strict environmental laws limiting drastic land conversations. Thus, the 2005-time period is representative of the current status of ES provision. All ES were assessed at the landscape scale and mean values were calculated for each municipality and ecoregion within the case study sites.

### ES trend development and upscaling

To assess the spatiotemporal characteristics of each ES in the different case studies, we calculated the changes in ES supply from the first to the last time period. The trends derived in this way for each service and ecoregion were then related to Zimmermann et al. (2010) LULC trajectories and extrapolated throughout the Alps. This was performed by generating an Alpine-wide map of ecoregions based on a digital elevation model (DEM) and the AgST classification. From the DEM, we derived a continuous map where each single pixel was allocated to a specific ecoregion according to its altitudinal zone and slope characteristics. Next, the most likely LULC-trajectory according to the prevailing AgST type (Tappeiner et al. 2003) was assigned to each pixel of an ecoregion. At that point, the ES trends could be related to the LULC trajectories using the same spatiotemporal unit. This procedure enabled us to transfer ES maps from the case study level to an Alpine-wide level and to derive continuous trend maps for each service. Online Resource 1 contains an overview of the LULC composition at different time periods and the main LULC trajectories derived from that data pool.

In our analyses, most non-agriculturally dominated ecoregions (those covered mainly by forests, vegetation-less nival belts, and near-natural alpine grasslands) showed no significant changes over the 150-year period (for example, forested land in 1850 in most cases resulted in forested land also in 2005) and were therefore excluded from subsequent analyses.

### Cluster analysis of ES trends

In the final step, the ES trends were analyzed from a geospatial perspective in order to uncover reoccurring statistical characteristics in the datasets. To accomplish this, we first performed a spatial pattern analysis for each ES trend map using the Moran’s I measure. Then ES bundles were identified based on the continuous ES trend maps by performing a *K*-means cluster analysis. Using the ‘no spatial constraints’ parameter to define the spatial relationship among features enabled us to group features that were not geographically close to each other. ES bundles were then mapped to show their spatial distribution over the Alpine mountain range and plotted in rose diagrams. All analyses were performed in ArcGIS Pro using the Spatial Analyst extension (ESRI 2014).

## Results

### ES trend maps

**Fig. 3:**
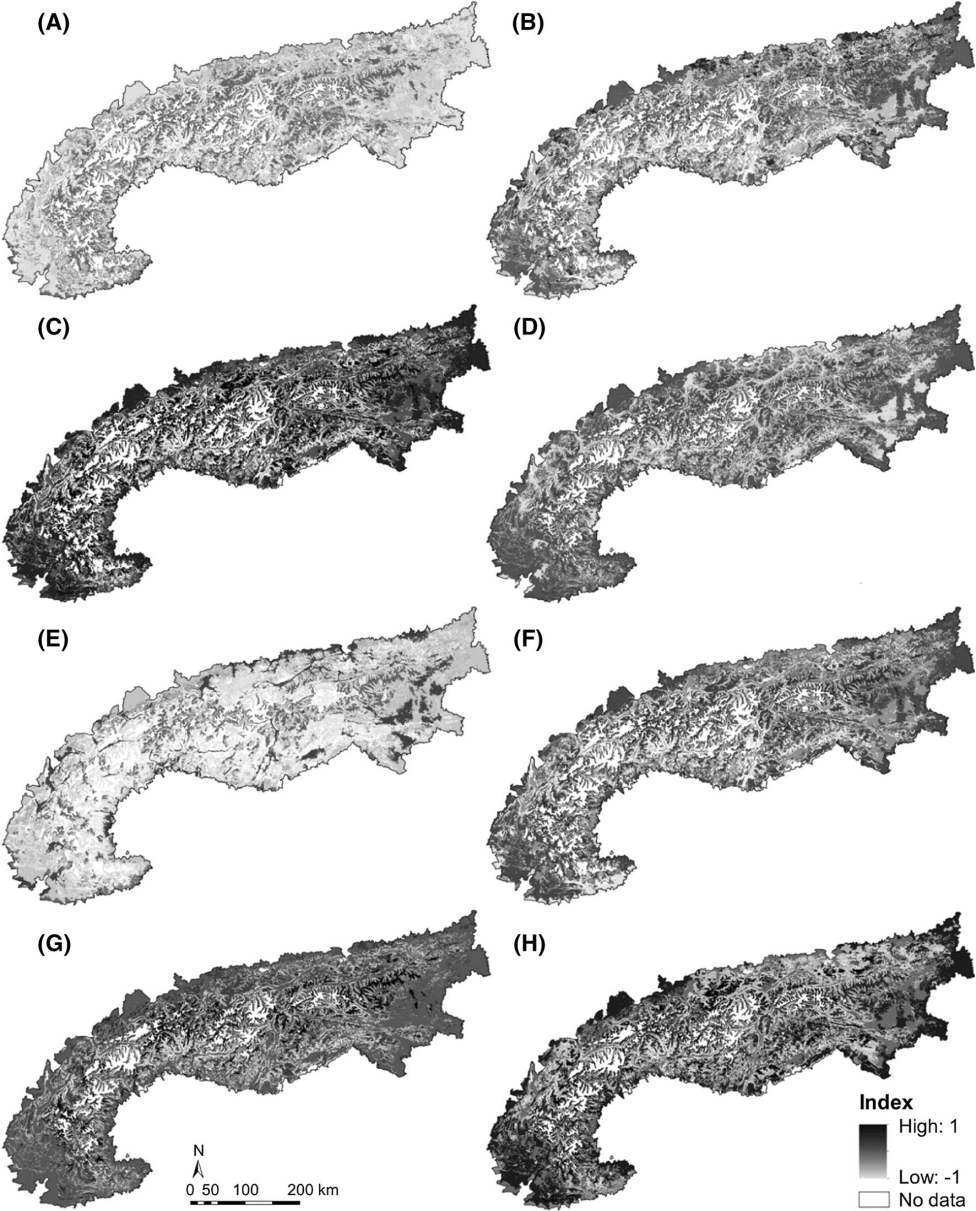
Spatial pattern of the changes in ES supply between 1850 and 2005 based on LULC trend maps (Zimmermann et al. 2010) at 250 m × 250 m resolution: **a** cultivated crops, **b** green biomass, **c** climate regulation, **d** soil erosion control, **e** pollination, **f** aesthetic value, **g** recreation and **h** mushroom picking. All maps are standardized to their highest occurrence (either positive or negative) on a scale between −1 and 1. *Positive values* indicate an increase in ES supply over time, and *negative values* indicate a decrease

To link the impacts of LULC change to ES provision at an Alpine-wide scale, we related the observed ES trends for the period 1850–2005 to LULC trends in the Alps. These maps provide spatially explicit information on potential ES hotspots and coldspots for each service (Fig. 3a–h). The observed land use dynamics have led to a modest increase in most ES values over the past 150 years. However, the impacts have altered the specific services to different degrees. Agricultural provisioning ES decreased over a large part of the Alps, especially in areas where land use transitions have led to abandonment of pastures and meadows or to alternative farming options. Only areas with higher production rates have continued to be actively managed. Climate regulation primarily benefited from general forest regrowth, especially after 1955 (Fig. 3c), and increased all over the Alps. Soil erosion control also improved especially on mountainsides, because of the steady increase in permanent plant cover. Pollination, on the contrary, defined as the potential contribution of natural habitats to provide the specific ES for pollination-dependent crops, largely decreased due to a reduction in cultivated crops in large parts of the Alps. A positive trend was only detected on some valley floors with favorable topo-climatic conditions (Fig. 3e). Cultural ES, namely aesthetic value, mushroom picking and recreation, generally increased over the entire period covered by this study, primarily benefiting from the abandonment of grasslands of high land use intensity. However, this process has been impeded by forest regrowth in recent decades (Fig. 3f–h). More generally, our results emphasize that ES sources can be found in landscapes that are characterized by a mixture of managed and unmanaged ecosystems, typically situated on mountainsides rather than on human-influenced valley bottoms and Alpine foothills.

### Spatial pattern

All ES maps were spatially clumped rather than randomly distributed in the landscape (Table 3). Although we found similarities between different types of ES, the spatial autocorrelation analysis revealed distinct types of spatial pattern for the different ES types. Provisioning ES tended to be clustered on the flattest areas in the fertile valley floors, while regulating ES were grouped in areas dominated by forest and a high degree of plant cover. Cultural ES showed spatial clustering in specific areas prone to recreational activities, such as extensive grasslands or forests.

**Table 3:** Moran’s I and related co-variables for testing spatial autocorrelation for each of the eight ecosystem services

Moran’s I		*z*-score	*p* value
Provisioning services			
Cultivated crops	0.78	8004.93	<0.0001
Green biomass	0.73	7517.58	<0.0001
Regulating services			
Climate regulation	0.83	8600.03	<0.0001
Soil erosion control	0.88	9076.63	<0.0001
Pollination	0.81	8315.41	<0.0001
Cultural services			
Recreation	0.75	7726.62	<0.0001
Aesthetic value	0.84	8675.92	<0.0001
Mushroom picking	0.66	6812.06	<0.0001

### ES bundle analysis

**Fig. 4:**
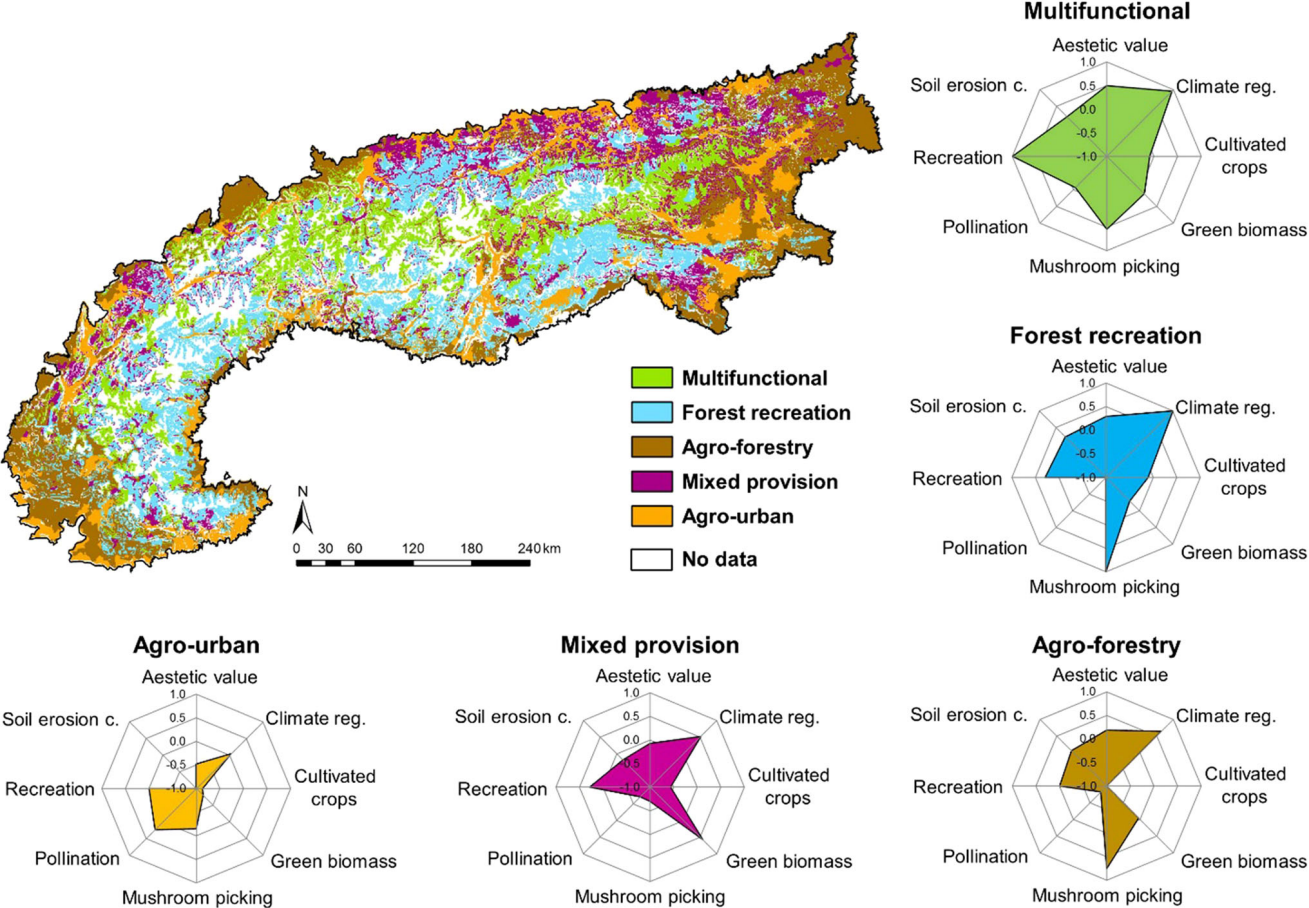
Spatial distribution of ES bundles produced by a cluster analysis. Rose diagrams indicate specific ES contributions within bundle types. *White surfaces* represent areas where no LULC trend was available (in general either not agricultural usable or forested areas)

By applying a statistical cluster analysis using the *k*-means algorithm on our ES trend maps we were able to identify five types of bundles (Fig. 4), representing the main pattern of ES provision in the Alps. For easier interpretation and discussion of the results, we named the bundles based on their main characteristics: *Multifunctional, Forest recreation, Agro*-*forestry, Mixed provision,* and *Agro*-*urban*.

The *Multifunctional* bundle is characterized by an increase in mean ES values for most services (except pollination and crops). It developed in areas with stable agricultural activities in favorable locations (mainly grassland farming) and was typically accompanied by natural reforestation processes on formerly abandoned land.

The *Forest recreation* bundle clustered ES linked to forests, such as climate regulation, recreation, and mushroom picking. All other ES were either decreasing or not present in the bundle. It typically appeared close to urban settlements on smooth hillsides and terraces.

The *Agro*-*forestry* bundle also drew on the natural reforestation and land abandonment processes observed over large parts of the Alps, but to lower degree. It differed by appearing also in lowland regions where grassland and livestock farming still played an important role (stable green biomass values). All other ES were either average or decreasing.

The *Mixed provision* bundle was characterized by a high presence of all three types of ES (provisioning, regulating, and cultural ES), expressed in above-average values in green biomass, climate regulation and recreation, and was typically located in the foothills of the Alpine mountain range.

The *Agro*-*urban* bundle was characterized by a decrease in most ES at lower elevations. Only pollination (along with stable climate regulating) had positive trends, mainly due to changes in agricultural activities (shift from grassland or annual crops to specialized permanent crops). Urbanization might have had an impact on this service bundle, as it appeared mostly near urbanized areas and some of the cultural ES had stable mean values (recreation).

## Discussion

In our study, we build on the well-known links between LULC and ES supply (Balthazar et al. 2015; Bürgi et al. 2015b) to address a major knowledge gap concerning the development of cross-border ES assessments over long timeframes (Dallimer et al. 2015; Renard et al. 2015; Tomscha et al. 2016). We combined the key findings of two scientific studies to advance the understanding of ES interactions under long-term LULC change (Tomscha and Gergel 2016). The first study, by Egarter Vigl et al. (2016), used a historical approach to derive the pattern of multiple ES for a set of representative case studies in the Alps. We adapted this approach by integrating it with a set of new ES indicators and case studies to increase its robustness. We aimed specifically to cover most of the variety in ES patterns for both the spatial and temporal components. The second work, by Zimmermann et al. (2010), identified a distinct number of LULC trajectories designed to represent the most important landscape dynamics of the past 150 years in the Alps. We used these results to develop a simple ES transfer function to scale ES trends from a landscape to an Alpine-wide level. The combined use of these data pools enabled us to gain further insights into the interactions between LULC change and the ES functionalities provided by mountain landscapes (Fu et al. 2015).

Over the past 200 years, anthropogenic activities have contributed heavily to reshaping the landscape, reflecting the most important societal, economic and technological transformations (Jespen et al. 2015). Most of these changes, especially in mountain regions, can be attributed to attempts to increase agricultural productivity, mainly through land intensification on fertile valley floors or on terraces (Bürgi et al. 2015a). However, these processes were often accompanied by land abandonment processes in unfavorable locations (Tasser et al. 2007) and urban sprawl (Antrop 2004). The direction in which a landscape develops can have direct impacts on the provision of underlying ES (Fu et al. 2015). According to Baral et al. (2013), different landscape compositions provide different sets of ES. Agricultural landscapes, for example, are typically prone to provisioning ES, such as cultivated crops or forage rather than to cultural ES (Power 2010), while forests tend to provide more regulating services, such as carbon sequestration or soil erosion control (Raudsepp-Hearne et al. 2010; Mäler et al. 2013).

### Ecosystem service trend mapping

ES trend mapping, as proposed in this paper, has been demonstrated to be valuable for representing changes in ES provision in a spatially explicit manner on the basis of long-term LULC trajectories. Producing ES trend maps allows easy identification, visualization and communication of the most important ES hotspots and coldspots and facilitates understanding of ES functioning and interconnection (Nemec and Raudsepp-Hearne 2013). Analyzing both ecological and social processes across administrative units allowed us to identify shared ES supply and trade-offs between separate regions in the Alps. Political boundaries rarely coincide with ecological ones and most species migrate freely across them, providing ES irrespective of jurisdictional boundaries. Thus, spatiotemporal analyses employing supranational datasets over large geographic areas are essential to managers and policy makers, as they account for the drivers of change over time (Tomscha and Gergel 2016). Moreover, land management actions and policy decisions in one country can have direct impacts on ES, and the related human well-being, in another (López-Hoffman et al. 2009).

From an Alpine-wide perspective, our results emphasize that the functionality of mountain landscapes depends on the regional ecological and socioeconomic conditions. Provisioning ES, especially annual crops and grasslands, suffered from land conversion and land abandonment processes, mainly as a consequence of changes in agricultural productivity (mechanization, synthetic fertilizers), globalization, subsidies and the availability of alternative forms of income (Van Zanten et al. 2014). These processes typically occurred on the most disadvantageous land parcels first, which were often difficult to access and cultivate. Regulating ES have benefitted from the steady increase in forested area over the past decades; an increase mainly driven by forest regrowth on former extensively managed grasslands or alpine pastures. The connection between forested LULC and regulating ES is found in a range of environments and is extensively documented in other studies (Raudsepp-Hearne et al. 2010; Mäler et al. 2013). Forest regrowth implies a potential increase in forest provisioning services such as timber production. Regions prone to cultural ES are often either close to urban settlements (Dallimer et al. 2015) and benefit from good accessibility, or are those of exceptional beauty (Schirpke et al. 2013). For the Alps, such sites can be found either in the populated valley bottoms and lowlands or on mountain peaks that provide adequate tourist infrastructure. Hence, cultural ES typically suffer from urban sprawl and agricultural intensification in fertile lowlands (mainly permanent cultivation), but benefit from more extensive agricultural use.

ES trend maps are mainly intended to demonstrate the tendencies of ES, rather than to precisely assess ES changes in biophysical terms or to predict future developments. The uncertainties in the driving forces are simply too great to derive valuable predictions for such a large geographic extent (Lawler et al. 2014). In light of an increasing need for ES assessment approaches at a transnational level and over a long time period (MA 2005; Maes et al. 2011), the present study could serve as a preliminary survey for regions where reliable or comprehensive data is lacking. Knowing how to link landscape changes to multiple ES provision enables us to estimate the most likely trajectories of ecological functioning and is essential for balanced landscape management at all spatial and temporal scales.

### Ecosystem service bundles

ES provision did not only show multiple linkages with LULC change, but also exhibited dynamic relationships with other ES in a spatiotemporal manner. This allowed us to define different groups of services (bundles) with similar ES patterns (Bennett et al. 2009). According to other studies, these bundles often represent the main LULC categories that underpin ES patterns, such as forest, agricultural land or urban settlements (Turner et al. 2014). In our study, we identified five bundles that were strongly linked to the LULC trajectories that we employed for the ES trend mapping. For terminology we relied, where possible, on previously published work to facilitate comparison between the different studies (Turner et al. 2014; Queiroz et al. 2015; Yang et al. 2015).

The *Multifunctional* bundle type generally had the most diverse collection of ES trends over time and was characterized by a mosaic of managed forest, grassland and alpine pastures. The combination of these three categories guaranteed a high supply of provisioning, regulating and cultural services. The *Forest recreation* bundle and *Agro*-*forestry* service bundle were dominated by increasing forest. Hence, they showed the best trends for both regulating and cultural ES, emphasizing a strong link between forests and specific types of cultural ES (Renard et al. 2015). The *Mixed provision* bundle usually provided at least one ES for each of the service types (provisioning, regulating, and cultural), and it was closely related to the *Multifunctional* bundle. We found that some peri-urban areas were particularly important for specific provisioning and cultural ES, reflected in the *Agro*-*urban* service bundle. However, the dynamics of ES in the European Alps, represented by these five bundles, does not align with conceptual models representing the variations of ES supply along a land use-intensity gradient as proposed by Braat and Ten Brink (2008) and De Groot et al. (2010). Rather it depends on the combination of the different socio-ecological variables, such as landscape history and topo-climatic conditions, and on the location specific interactions of these characteristics (Crouzat et al. 2015). Moreover, social legacies, as described by Locatelli et al. (in revision) for specific regions and landscapes in mountain areas, depict crucial and innovative components that have to be considered and studied further in order to advance in the understanding of coupled human-environmental systems.

### Methodological considerations

In this study, we combined data available from previous studies, which allowed us to provide a spatiotemporal approach for ES trend mapping and clustering for a large geographic area. However, this also had some limitations that have to be considered when drawing conclusions from the present work. First, the selection of the case studies relied on a study by Tappeiner et al. 2003 that classified the Alpine arc into eight AgST using mainly socioeconomic data representative for the time period (1980–2000). This leads to some limitations in our approach in terms of validity for the entire period under study (1850–2005). However, we considered this aspect of minor importance, as the main land use patterns were very similar throughout the Alps until the mid-1960s (Tasser et al. 2007). It was only after this decade that more significant changes in land use regimes influenced landscape composition (Jepsen et al. 2015). Second, the subsequent upscaling from case study to an area-wide level, using an ES transfer function derived from topo-climatic and land use characteristics, cannot completely account for all the complex socioecological dynamics occurring in Alps. It does, however, form a solid basis for extrapolating broad ES trends throughout the Alps, especially given the inclusion of important historical developments on the landscape. Third, ES trends rely on a data pool available at four points in time. The dynamics occurring between these periods could not be considered. Fourth, the study used data sets from different sources and with different levels of resolution and quality. Hence, for some ES, we might have under- or over-estimated the variations in the provision over time. Fifth, we used a limited set of indicators to analyze the dynamics within and between ES types and LULC, and thus we might not have captured important factors, such as shifts in societal preferences, soil properties and climate change. However, we have attempted to provide an analysis that is representative of some of the most crucial ES in mountain regions. A full analysis of the multiple interrelationships on an Alpine-wide level was not the scope of this study, as an adequate data pool for such an analysis does not exist. Thus, we concentrated on the identification of the main Alpine-wide ES trend developments. These developments can help us to better understand and manage the full complexity of ES in mountain regions.

## Conclusion

This paper traced the history of ES developments and interrelationships to explore the multiple functionalities of mountain landscapes. Our main aim was to promote a spatiotemporal approach that addresses ES management from two perspectives: first, from a transnational point of view with no boundary constraints for ecological functioning, and second, from the perspective of reoccurring socioecological patterns across the landscape, presented as ES bundles. ES trend maps produced for the entire Alpine region provide a valuable tool to spatially demonstrate the long-term influences of LULC changes on ES development and to highlight locations where ES hotspots or coldspots are likely to appear. Understanding the diversity of spatiotemporal patterns in mountain terrain is important for balancing land management decisions at multiple administrative levels for both intensively used land as well as legally protected areas. Moreover, our results can promote the integration of ES assessments into environmental management policies and thereby address the requirements of the EU Biodiversity Strategy. It is essential to foster this integration, as ES bundles are typically the result of long-term socioecological relationships occurring across the landscape. Therefore, managing for the underlying relationships of these human-natural systems through adequate policies and measures may provide the best opportunity for maintaining and enhancing the functionalities of mountain landscapes into the future.

## Electronic supplementary material

Below is the link to the electronic supplementary material.

Supplementary material 1 (DOCX 705 kb)
