# Supplementary material for: Using land use/land cover trajectories to uncover ecosystem service patterns across the Alps
Source: Reg Environ Change. 2017 Mar 11;17(8):2237–50. doi: 10.1007/s10113-017-1132-6 (PMC6959402; doi:10.1007/s10113-017-1132-6)
Supplement: Supplementary file 1 — Supplementary material 1 (DOCX 705 kb) [file 10113_2017_1132_MOESM1_ESM.docx]

**Online Resource 1:**

1. **LULC trajectories:**

The LULC trajectories, used in this paper as the starting point for the ES upscaling procedure and ES trend development, were retrieved from Zimmermann et al. (2010). The figure below summarizes the composition and spatial distribution of the 6 main trajectories found in that analyses.

**
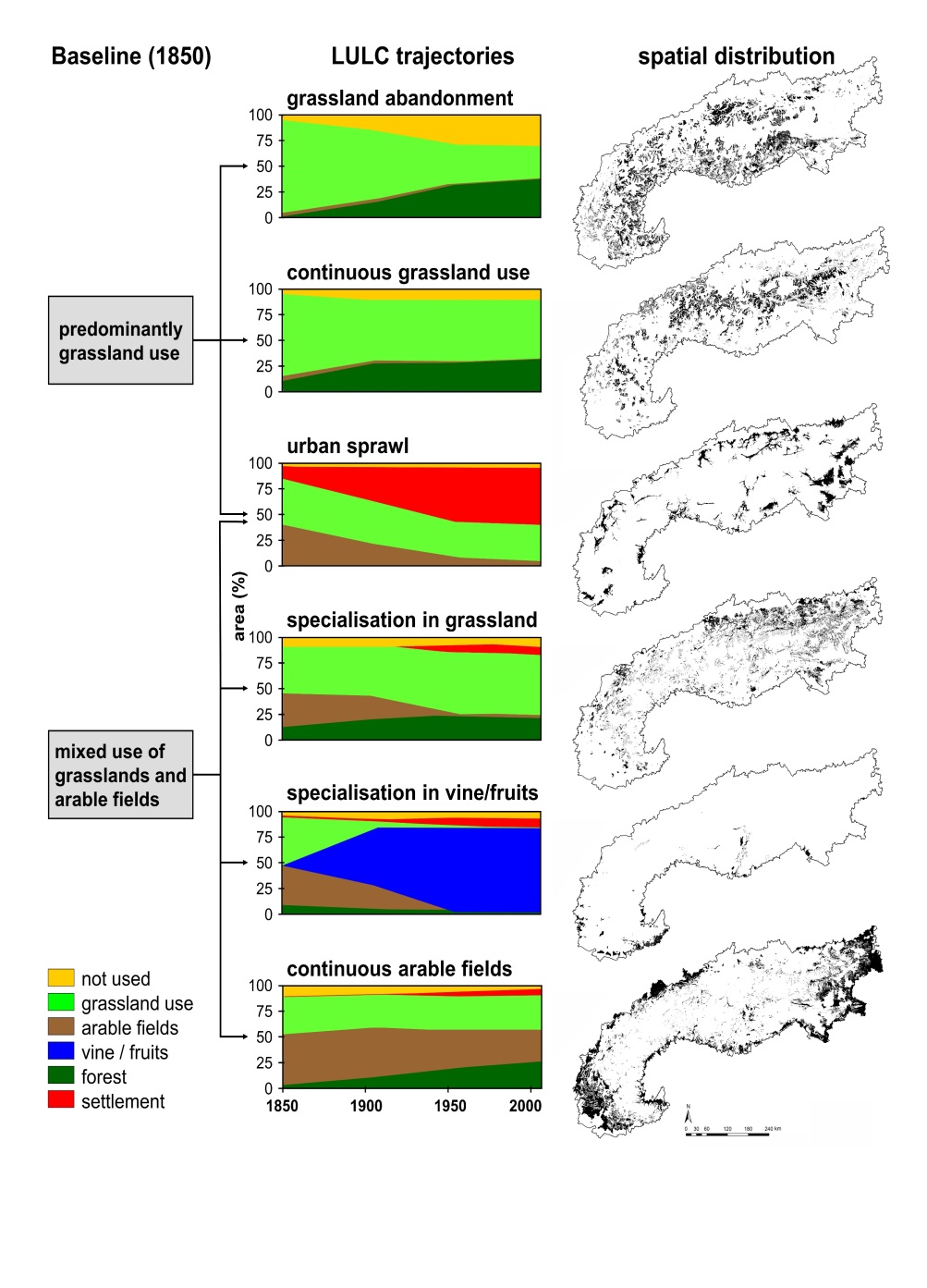
**

1. **ES assessment:**

We performed a spatiotemporal analysis on a number of eight ES, including two provisioning ES (Cultivated crops, Green biomass), three regulating ES (Climate regulation, Soil erosion control, Pollination potential) and three cultural ES (Aesthetic value, Recreation, Mushroom picking). For all ES we calculated area-wide mean values per ha. We referred to the CICES 4.3 classification for ES terminology.

**Cultivated crops**

The cultivated crop ES was estimated based on the number of man hours (h ha−1) deployed to acquire the basic commodities produced in one hectare of a specific crop. This non-monetary indicator reflected both, variations in the national monetary systems (currency changes and/or monetary de- or inflation) and the developments in agricultural productivity. The data inputs for the analysis were based on an extensive literature review, on records from the historic [chronicle](http://dict.leo.org/ende/index_de.html#/search=chronicle&searchLoc=0&resultOrder=basic&multiwordShowSingle=on)s of the Hapsburg Monarchy and the Bavarian Empire and on data from national statistical institutes (Klose and Jungmann-Stadler 2006; Trapp 1999; Pies 2008; Mattes 1929; Rauser 1980).

**Green biomass**

The green biomass ES was estimated based on the productivity of different types of permanent grasslands (Egger et. al. 2004, Tasser et al. 2012). The approach considered the length of growing season, derived as a function of elevation and climate conditions (Harflinger and Knees 1999), a topographical correction factor, depending on slope and aspect and the amount of summer mean precipitation.

**Climate regulation**

To assess the climate regulation ES we assigned to each land cover type the carbon pool (Mg C ha−1) according to aboveground and belowground phytomass (Mg ha−1) and C-stock (g g−1). The relevant datasets were derived from a literature review and our own measurements (Tappeiner et al. 2008; Schirpke et al. 2013a). For the carbon stocks of forests, we additionally considered the biomass density change at the different time periods (Patek 2013).

**Soil erosion control**

The estimation of the soil erosion control ES was based on a modified Universal Soil Loss Equation (USLE; Wischmeier and Smith 1978), where the root density was used for the stability factor, the slope angle for the topographic factor and the mean vegetation cover of the specific land cover types for the management factors (Schirpke et al. 2013a). Mean vegetation cover and root density were derived from our own measurements (Tasser et al. 2005; Tappeiner et al. 2008), while the slope angel was calculated from the digital elevation model.

**Pollination potential**

The pollination ES was defined as the capacity of natural ecosystems to provide the services to pollination depending crops (Maes et al. 2011). For each crop land use we calculated two probabilities: first, the crop dependency ratio based on the specific crop type (Klein et al. 2007) and second, the visitation probability as a regression between distance to natural habitat and visitation rate (Ricketts et al. 2008). The sum of these two variables were then assigned to the closest natural ecosystem.

**Aesthetic Value**

For the assessment of the aesthetic value we combined the results of i) a questionnaire based photo survey on alpine landscapes (Schirpke et al. 2013b), ii) a topographical visibility analysis (from DEM) and iii) a measure of landscape diversity (Shannon index) (Schirpke et al. 2013a). Each value was then related to the correspondent LULC category and summed up to a single value. Each of the three components were weighted equally.

**Recreation**

The recreational ES was expressed in ha capita-1 according to a study by Lauf et al., 2014 and defined as the sum of all recreational areas within a 5 km distance radius from urban settlements (Pouta and Sievänen 2001). We defined the following land uses suitable for recreational purposes: forests, abandoned land, water courses and grassland areas. To account for differences in land use intensity, we applied a correction factor for the different grassland types.

**Mushroom picking**

We considered mushrooming picking potential as a cultural ES according to Navarro and Pereira, 2012. The ES was calculated as the available area (in ha capita-1) that meet the following conditions: be either forested land cover or extensive grassland, have an altimetry not above 2000 m a.s.l., and a slope angle lower than 80% (Schirpke et al, 2014). Furthermore, a distance weighting factor was applied to all suitable picking sites, based on a gradual distance function from close by walking paths (<1km) (Yong and Diez-roux 2012).

1. **Statistical cluster analyses**

We performed a statistical cluster analyses using the K-means algorithm over all ES trend maps using ArcGIS Pro software (Esri, 2016). The table below summarizes the results of the analyses for each group and service.

| 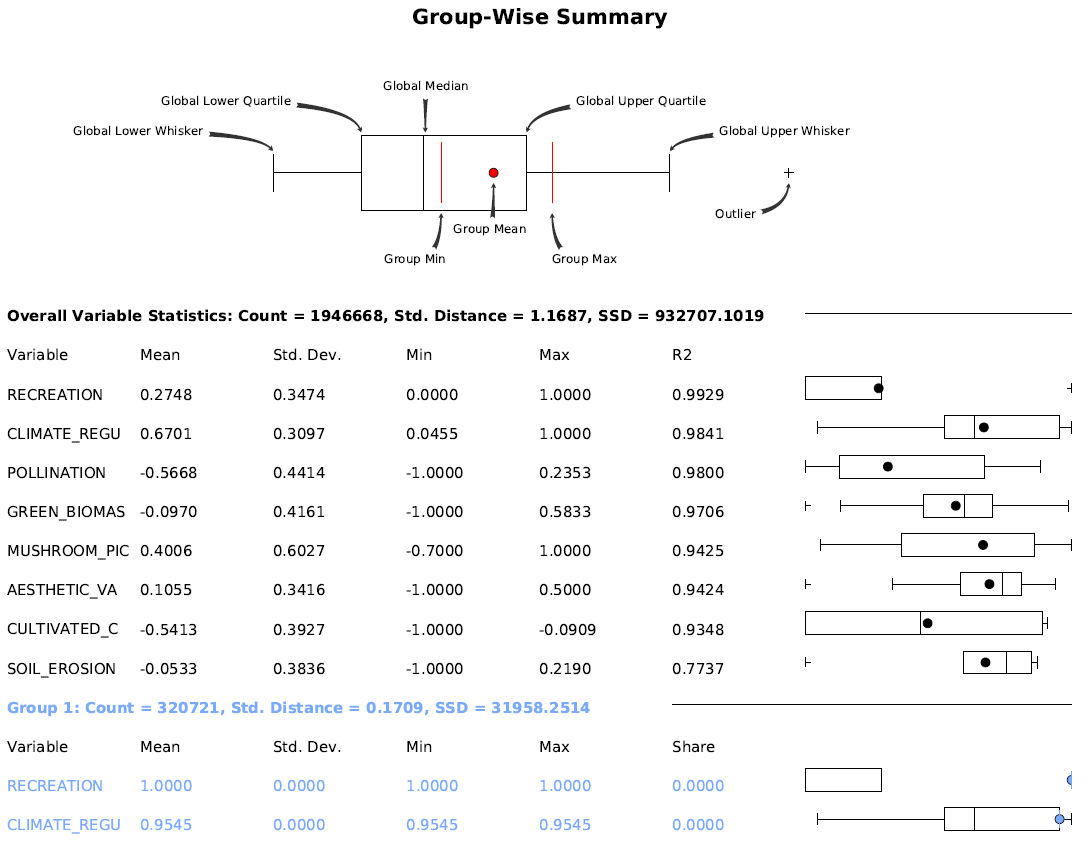 |
| --- |
| 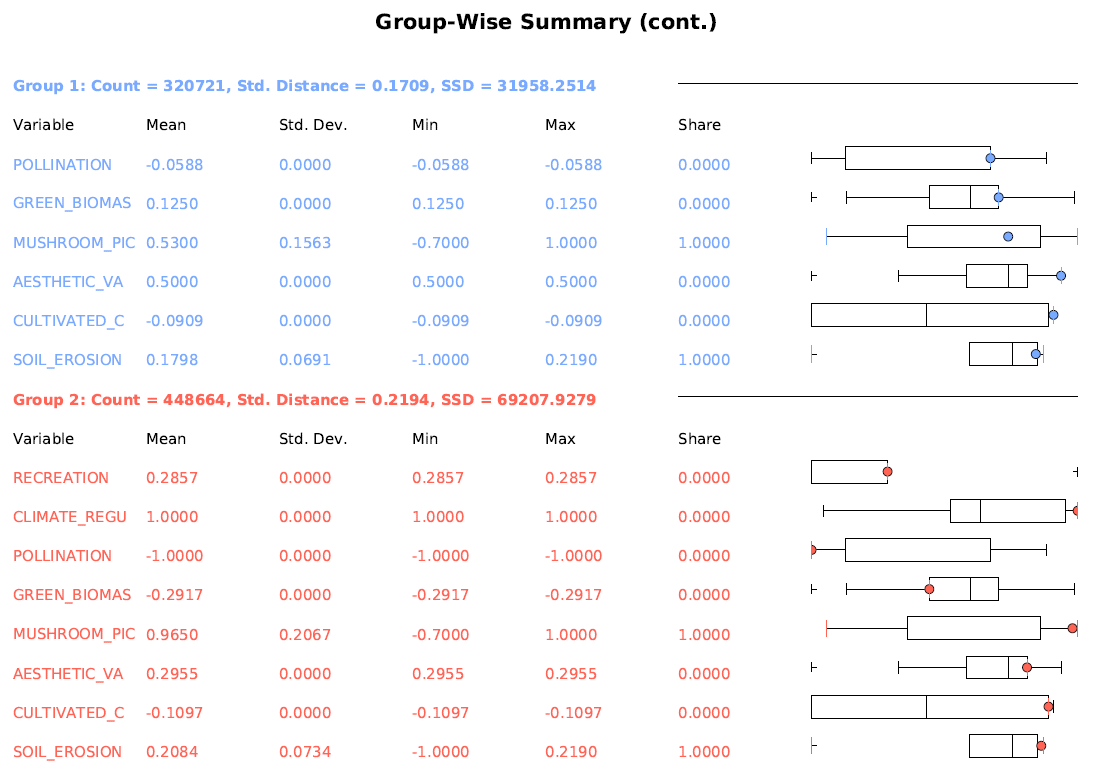 |
| 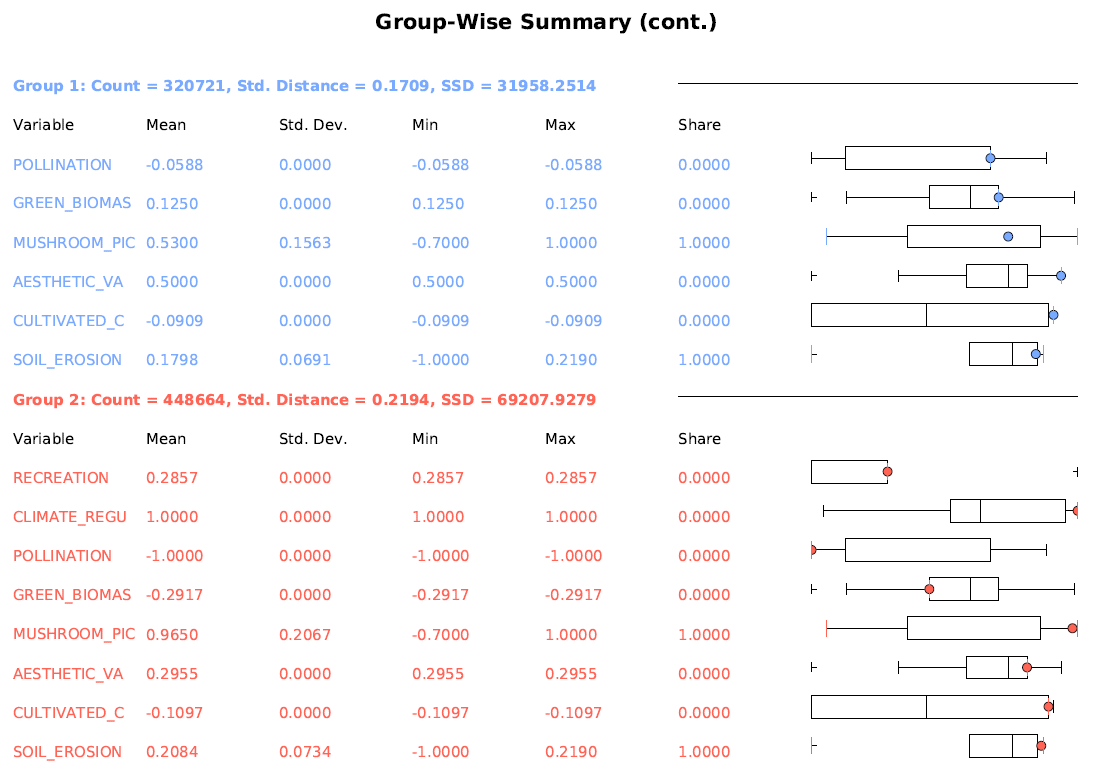 |
| 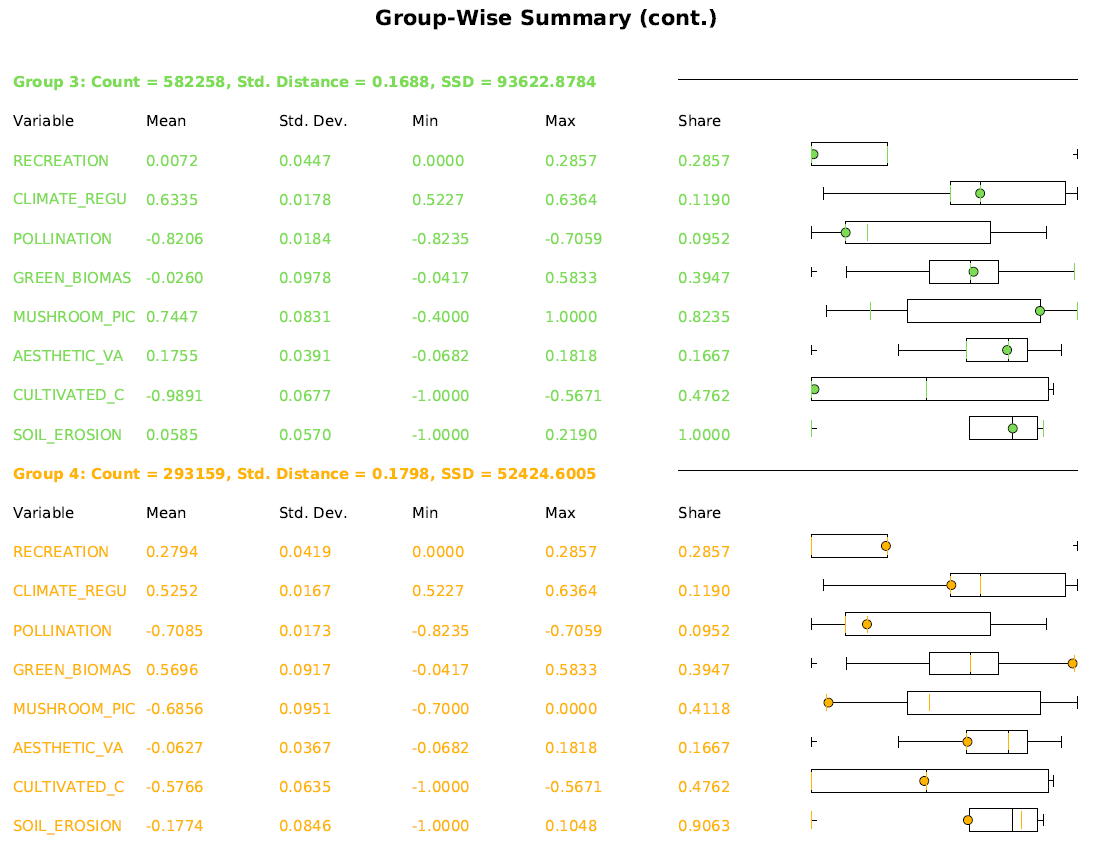 |
| 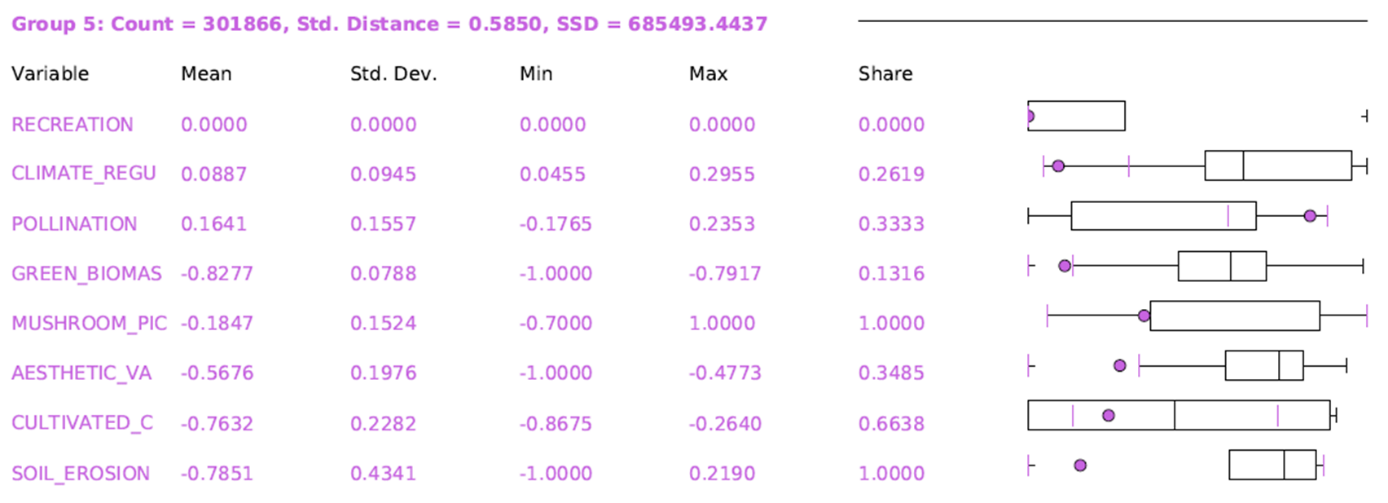 |

1. **Reference**

Lauf S., Haase D., Kleinschmit B., 2014. Linkages between ecosystem services provisioning, urban growth and shrinkage – A modeling approach assessing ecosystem service trade-offs. Ecological Indicators 42 (2014) 73–94.

Klose D, Jungmann-Stadler F (2006) Ko¨niglich Bayerisches Geld - Zahlungsmittel und Finanzen im Konigreich Bayern 1806–1918. Staatliche Münzsammlung, München

Mattes W (1929) Oehringer Heimatbuch. Verlag Hohenlohesche Buchhandlung Ferdinand Rau. Öhringen

Navarro L.M., Pereira H.M., 2012. Rewilding abandoned landscapes in Europe. Ecosystems, 15 (2012), pp. 900–912

Pies E (2000) Löhne und Preise von 1300 bis 2000. Abhängigkeit und Entwicklung über 7 Jahrhunderte. Verlag E. & U, Brockhaus, Wuppertal

Pouta, E. and Sievänen, T. (2001) Luonnon virkistyskäytön kysyntätutkimuksen tulokset -Kuinka suomalaiset ulkoilevat? (Results of the demand study). In Luonnon virkistyskäyttö 2000 (Summary: Outdoor recreation 2000) Sievänen, T. (ed.), 336pp, Metsäntutkimuslaitoksen tiedonantoja 802, 32-76, 195-196.

Rauser JH (1980) Waldenburger Heimatbuch. Aus der Ortsgeschichte von Waldenburg und Obersteinbach / Sailach 4

Schirpke U, Leitinger G, Tasser E, Schermer M, Steinbacher M, Tappeiner U (2013a) Multiple ecosystem services of a changing Alpine landscape: past, present and future. Int J Biodiv Sci Ecosyst Serv Manag 9:123–135

Schirpke U, Tasser E, Tappeiner U (2013g) Predicting scenic beauty of mountain regions. Landsc Urban Plan 111:1–12

Schirpke U., Scolozzi R., De Marco C., Tappeiner U., 2014. Mapping beneficiaries of ecosystem services flows from Natura 2000 sites. Ecosystem Services 9 (2014) 170–179.

Sievänen, T., Pouta, E. ja Neuvonen, M., 2004. Participation in Mushroom Picking in Finland. In Ito, T. & Tanaka, N. (eds.) Social Roles of Forests for Urban Population. Forest Recreation, Landscape, Nature Conservation, Economic Evaluation and Urban Forest. Japan Society of Forest Planning Press. p. 122-137.

Trapp W (1999) Kleines Handbuch der Münzkunde und des Geldwesens in Deutschland. Reclamverlag, Stuttgart

Yang, Y., & Diez-Roux, A. V. (2012). Walking Distance by Trip Purpose and Population Subgroups. American Journal of Preventive Medicine, 43(1), 11–19. http://doi.org/10.1016/j.amepre.2012.03.015
